# Ipsilateral and Contralateral Interactions in Spinal Locomotor Circuits Mediated by V1 Neurons: Insights from Computational Modeling

**DOI:** 10.3390/ijms23105541

**Published:** 2022-05-16

**Authors:** Natalia A. Shevtsova, Erik Z. Li, Shayna Singh, Kimberly J. Dougherty, Ilya A. Rybak

**Affiliations:** Department of Neurobiology and Anatomy, College of Medicine, Drexel University, Philadelphia, PA 19129, USA; nas29@drexel.edu (N.A.S.); ezl24@drexel.edu (E.Z.L.); sps84@drexel.edu (S.S.); kjd86@drexel.edu (K.J.D.)

**Keywords:** spinal cord, central pattern generator, neural circuits, genetically identified interneurons, V1 interneurons, neural control of locomotion, computational modeling

## Abstract

We describe and analyze a computational model of neural circuits in the mammalian spinal cord responsible for generating and shaping locomotor-like oscillations. The model represents interacting populations of spinal neurons, including the neurons that were genetically identified and characterized in a series of previous experimental studies. Here, we specifically focus on the ipsilaterally projecting V1 interneurons, their possible role in the spinal locomotor circuitry, and their involvement in the generation of locomotor oscillations. The proposed connections of these neurons and their involvement in different neuronal pathways in the spinal cord allow the model to reproduce the results of optogenetic manipulations of these neurons under different experimental conditions. We suggest the existence of two distinct populations of V1 interneurons mediating different ipsilateral and contralateral interactions within the spinal cord. The model proposes explanations for multiple experimental data concerning the effects of optogenetic silencing and activation of V1 interneurons on the frequency of locomotor oscillations in the intact cord and hemicord under different experimental conditions. Our simulations provide an important insight into the organization of locomotor circuitry in the mammalian spinal cord.

## 1. Introduction

The spinal cord of vertebrates, including mammals, contains neural circuits, generally referred to as central pattern generators (CPGs), that are capable of autonomous generation, patterning, and coordination of rhythmic neuronal activity involved in the control of locomotion [1,2,3,4,5,6,7,8,9,10]. The locomotor-related rhythmic activity, also called “fictive locomotion”, can be evoked *in vitro* in the isolated spinal cords of neonatal rodents by application of special combinations of neuroactive drugs [11,12,13,14,15,16], or electrical stimulation of specific brainstem or spinal regions, or sensory afferents [17,18,19,20,21,22,23]. The major characteristics of these locomotor-like oscillations include phase alternation (antiphase synchronization) in the activity of flexor-related and extensor-related motor outputs (flexor–extensor alternation) and in the activity of homonymous motor outputs from left and right hemicords (left–right alternation) [7].

The architecture of spinal circuitry and neural elements involved in generation and shaping of locomotor rhythmic activity remain poorly understood [5,6,7,9,10]. Earlier studies were limited due to the lack of clear spatial segregation of spinal neurons involved in different functions. Significant progress has been achieved in the last two decades with the development of novel genetic and molecular approaches allowing for the identification and classification of distinct spinal interneurons by transcription factor expression [9,24,25,26]. Results from selective genetic-based manipulations (ablation, disruption of synaptic transmission, hyperpolarization, or depolarization) suggested specific functions for some neuron types and their possible roles in the spinal locomotor circuitry [27,28,29,30,31,32,33,34,35,36,37,38]. The identified neuron types include the commissural interneurons (CINs), such as V0 (V0_D_ and V0_V_) [27,28] and V3 [29,30,31], and ipsilaterally-projecting V2a neurons [37,38] that are critically involved in the coordination of left–right rhythmic activities, as well as the ipsilaterally-projecting inhibitory V1 and V2b neurons involved in flexor–extensor alternation [32,33]. In addition to their roles in coordinating flexor–extensor activity, V1 neurons have been found to be involved in the control of the frequency of fictive locomotion [32,34,35,36,39].

Despite the obvious progress in spinal neuron identification, the existing information on connectivity and interactions between different types of neurons is very limited, even for those that have been genetically identified. In this case, further progress in the understanding of the organization and operation of spinal locomotor circuits can be achieved using computational modeling of spinal circuits. The developed computational models should be able to simultaneously reproduce multiple sets of experimental data, including the results of selective manipulations of identified neuron types performed in different laboratories under different experimental conditions. We have previously developed a basic model and continue updating, extending, and elaborating this model as new experimental results become available [31,40,41,42].

Recently, new experimental studies were performed to investigate the role of V1 neurons in spinal circuits [35,36]. These studies described the effects of optogenetic manipulations of V1 neurons on the fictive locomotor activity evoked in the isolated spinal cord and hemicord from neonatal mice. The main objectives of the present study were: (1) to test and update our model of the mammalian spinal locomotor circuitry based on these experimental data; and (2) to propose (and predict) the possible role of V1 interneurons in the spinal locomotor circuitry, their involvement in multiple ipsilateral and contralateral neuronal pathways in the spinal cord, and their contribution to the control of frequency of locomotor oscillations.

## 2. Results

Following recent studies of Falgairolle and O’Donovan [35,36], two experimental conditions were considered in our simulations. First, we modelled the conditions in which the locomotor-like activity was induced by neuroactive drugs in the whole lumbar cord and in a hemicord. Then, we simulated the conditions when the locomotor-like activity was evoked by brainstem stimulation. In both conditions, we simulated the effects of optogenetic stimulations producing either hyperpolarization (silencing) or depolarization (activation) of V1 neurons in the spinal network and compared our modeling results with the corresponding experimental data of Falgairolle and O’Donovan. Our goal was to test the proposed connectome of the spinal neural circuitry, provide reasonable explanations for the corresponding experimental results, and suggest the roles of V1 interneurons in the regulation of locomotor activity under different conditions.

### 2.1. Model Description

#### 2.1.1. Architecture of Spinal Circuits in the Model

The organization of locomotor circuitry in our model is based on network interactions among several populations of genetically identified (V0_D_, V0_V_, V1, V2a, V2b, and V3) and some unidentified (but suggested) spinal interneurons proposed in our previous modeling studies [31,40,41,42]. Specifically, the neuronal connectome of the model represents a combination of network architectures proposed in two our previous models [31,42].

**Figure 1:**
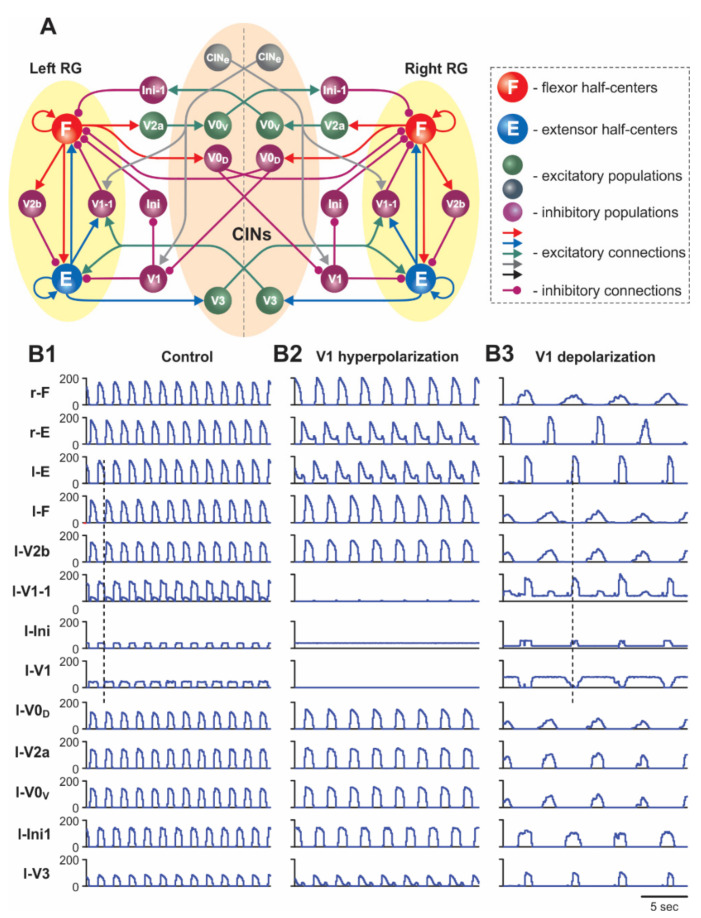
(**A**) Model schematic. (**B1**–**B3**) Model performance in control conditions (**B1**), after hyperpolarization of V1 neurons (**B2**), and after depolarization of V1 neurons (**B3**). Activity profiles of populations in this and the following figures are shown as average histograms of neuron activity (spikes/(N × s), where N is the number of neurons in a population; bin = 100 ms). In (**B1**–**B3**), activities of the F and E half-centers and left (l-) interneuron populations are shown at α = 0.17. The dashed line in (**B1**) indicates the beginning of left flexion, and in (**B3**) the beginning of left extension.

The schematic of the model is shown in Figure 1A. The model represents a bilateral structure that includes two rhythm generating neural networks (left and right RGs) interconnected by several commissural pathways mediated by specific populations of commissural interneurons (V0_D_, V0_V_, and V3 commissural interneurons, CINs). Each RG includes flexor (F) and extensor (E) half-centers. These half-centers represent populations of excitatory neurons with intrinsically bursting properties defined by the persistent (slowly inactivating) sodium currents ($I_{\mathrm{NaP}}$) [41,42]. These bursting properties are state dependent, so that with increases of excitation, the state of each neuron changes from silence to rhythmic bursting, and then to sustained tonic activity. In the bursting regime, the frequency of rhythmic oscillations increases with an increase in neuron excitation. As in our previous models [31,40,41,42,43,44], we implemented an asymmetric RG organization in which only F half-centers operate in the bursting regime, whereas the E half-centers if isolated operate in the tonic mode and exhibit rhythmic activity because of the phasic inhibition from ipsilateral F half-centers (for details, see [41]). The F and E half-centers on each side mutually inhibit each other through inhibitory interneurons (V2b and V1-1, Figure 1A, see also [42]), thus supporting flexor–extensor alternation.

Commissural interactions between the left and right RGs are organized similarly to our previous models [31,41,42,43,44,45] (Figure 1A). Specifically, the V0_D_ and V0_V_ CINs provide mutual inhibition between the F half-centers of the left and right RGs and support the left–right alternating activity, and the V3 CINs mediate mutual excitation between the E half-centers of the left and right RGs and provide synchronization of the left and right RG activities in some specific experimental conditions [31].

#### 2.1.2. Two Functionally Distinct Types of V1 Populations in the Model and Their Connectivity

**Figure 2:**
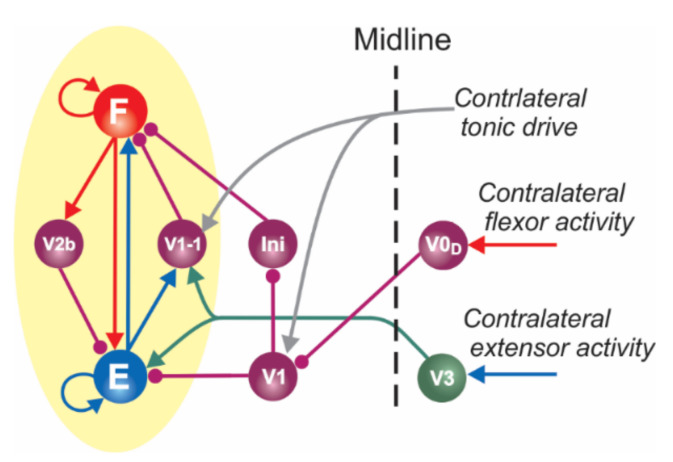
Two functionally distinct populations of V1 neurons in the model and their connectivity.

Following our previous modeling studies [42], the present model includes two types of functionally distinct populations of V1 neurons, as highlighted in Figure 2. One type, designated as the V1 population, operates in both left and right circuits. The left and right V1 populations are involved in disinhibition of the ipsilateral flexor half-center (F) through inhibition of the corresponding inhibitory Ini population suppressing this half-center (see Figure 1A and Figure 2) and direct inhibition of the ipsilateral extensor half-center (E). The activation of these V1 populations promotes an increase of oscillation frequency. The other type of V1 population, designated in the model as V1-1, also operates in the left and right circuits. Each V1-1 population mediates inhibition of the ipsilateral F half-center by the corresponding E half-center and hence participates in the flexor–extensor alternation on each side.

**Figure 3:**
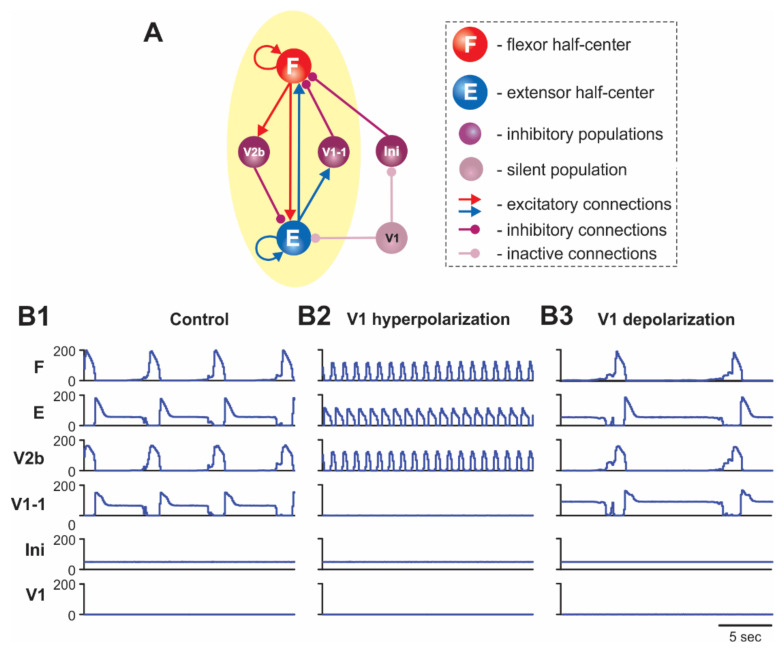
(**A**) Schematic of the hemisected model. (**B1**–**B3**) Model performance in control conditions (**B1**), after hyperpolarization of V1 neurons (**B2**), and after depolarization of V1 neurons (**B3**). Activities of all populations are shown at α = 0.2.

As shown in Figure 1A and Figure 2, the ipsilateral V1 and V1-1 populations in the model receive contralateral tonic excitation, although the input to the V1 populations is much stronger than that to the V1-1 populations. Therefore, the activity of left and right V1 populations strongly depends on the contralateral drive, whereas the activity of left and right V1-1 populations is mostly defined by the activity of the corresponding ipsilateral E half-centers. In addition, each (left and right) V1 population receives contralateral inhibition during contralateral flexion (from the contralateral V0_D_ CINs). These connections are necessary to make the activity of V1 populations rhythmic and synchronized with the contralateral E (ipsilateral F) activity. The rhythmic activity of V1 populations results in the rhythmic inhibition of the ipsilateral E half-centers, allowing the model to maintain rhythmic extensor activity in the cord in the absence of (ablated) ipsilateral V2b inhibition ([32]; see details in our previous paper [42]). In contrast, left and right V1-1 populations receive excitation from the contralateral V3 CINs. These connections were incorporated in the model to reproduce the results of optogenetic activation of contralateral V3 interneurons [31]. All contralateral influences including contralateral drives disappear in the “hemicord model” to simulate the effects of hemisection (Figure 3A).

### 2.2. Simulation of Drug-Induced Fictive Locomotion

To simulate fictive locomotion induced in the spinal cord by neuroactive drugs, we used a model version shown in Figure 1A for the “intact cord” and Figure 3A for the “hemicord”. The effect of drug application was modelled as an increase in the level of excitation of all neurons in the model by elevating (depolarizing) their leakage reversal potentials (see Methods and our previous publications [31,41,42]). The results of simulations of drug-induced locomotion in the intact cord and hemicord are shown in Figure 1B1 and Figure 3B1, respectively. The model of intact cord exhibited alternating oscillations in the flexor and extensor RG half-centers on each side of the cord as well as alternating activities in the homonymous left and right half-centers (Figure 1B1).

### 2.3. Changes in the Drug-Induced Locomotor Oscillations after Simulated Hemisection

Previous studies on isolated cords from neonatal mice have shown that drug-induced fictive locomotor activity can be evoked in isolated mouse hemicords obtained by midsagittal cord transections [32,35,36,46,47]. The common observation from these studies is that the frequency of drug-induced oscillations in the hemicord is usually significantly less than that in the intact cord. However, to our knowledge, the mechanisms of this frequency reduction after hemisection remain unknown.

In our model, after simulated hemisection, the V1 population loses the contralateral excitation and becomes silent (Figure 3A). Silencing of the V1 population eliminates its inhibition of the inhibitory Ini population, which therefore maintains tonic activity (due to simulated drug application) (Figure 3B1). The tonic activity of the Ini population provides constant inhibition of the F half-center, resulting in a reduction in the frequency of the locomotor oscillations (compare Figure 3B1 with Figure 1B1).

**Figure 4:**
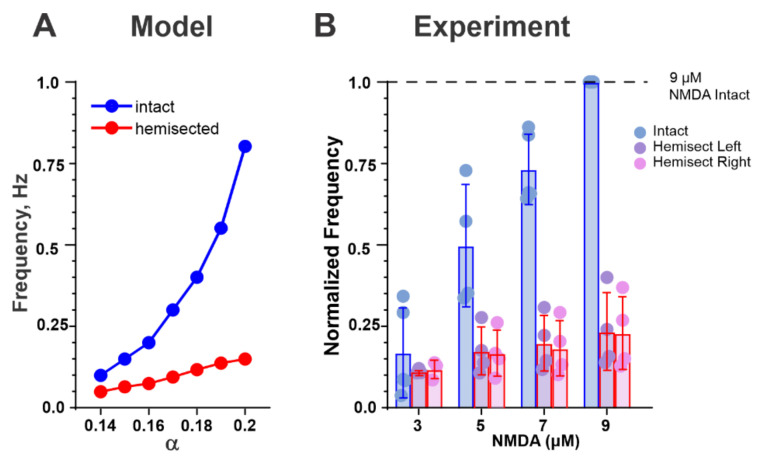
(**A**) Dependence of oscillation frequency on the parameter α in the intact (blue) and hemisected (red) models. (**B**) Normalized frequency of the locomotor-like activity recorded from ventral roots versus NMDA concentration in isolated neonatal spinal cords before (intact, blue bars and data points) and after hemisection (red bars). The concentration of 5-HT was constant (8 μM). The purple and pink data points represent the left and the right hemicord, respectively.

Figure 4A shows how the frequency of drug-induced oscillations in both the intact and hemicord models changes with an increase of neuronal depolarization, simulating an increase of drug concentration. Our simulations predicted a monotonic increase in oscillation frequency in both the intact and hemisected cords with a steeper frequency increase in the case of the intact cord. To test this prediction, spinal cord preparations from neonatal mice were used to measure changes in the frequency of locomotion induced by NMDA and 5-HT, with changes in NMDA concentration before and after midsagittal hemisection (Figure 4B). The results of these studies were qualitatively consistent with our modeling predictions, providing additional validation to our models.

### 2.4. Simulation of Brainstem Stimulation-Evoked Fictive Locomotion

**Figure 5:**
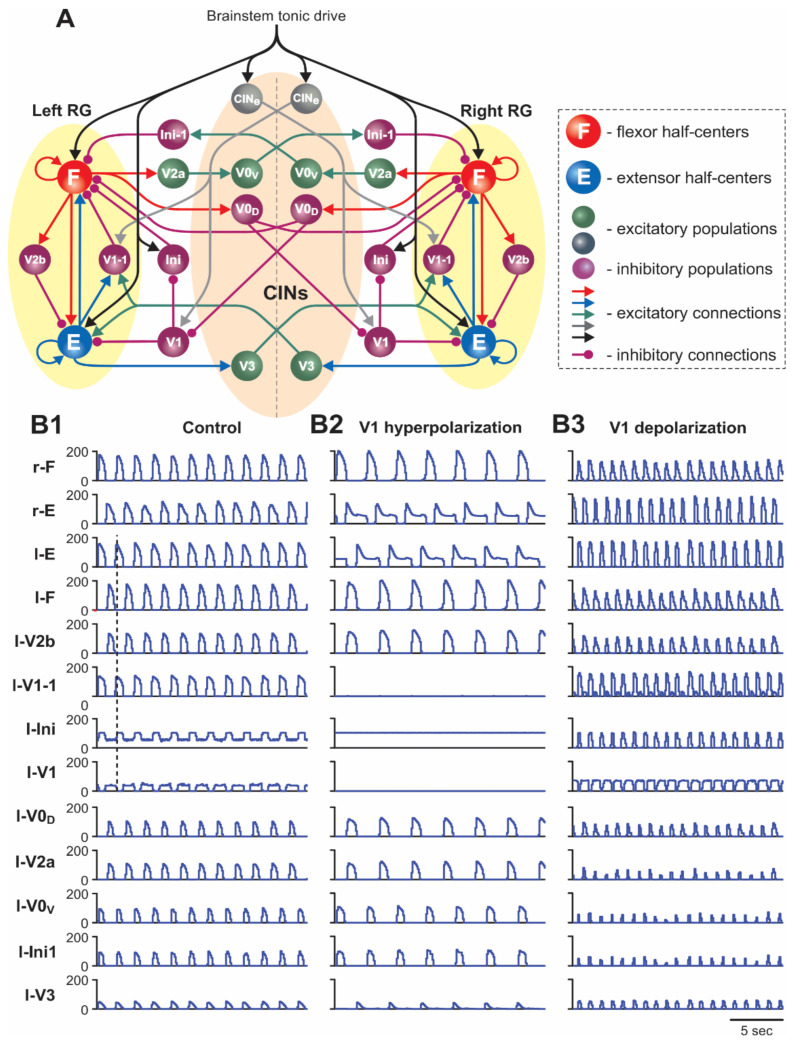
(**A**) Schematic of the model version incorporating the brainstem drive. Thick black lines show the distribution of the excitatory tonic drive from the brainstem to specific neuron populations. (**B1**–**B3**) Model performance in control conditions (**B1**), after hyperpolarization of all V1 neurons (**B2**), and after depolarization of all V1 neurons (**B3**). In (**B1**–**B3**), activity profiles of the F and E half-centers and left (l-) interneuron populations are shown at α = 1. The dashed line in (**B1**) indicates the beginning of left extension.

To simulate the fictive locomotor oscillations evoked by brainstem stimulation, a modified model version was used (Figure 5A). This model version had the same architecture of the spinal network. However, in contrast to simulations of drug-induced locomotion, the level of neuronal excitation was not changed. Instead, we incorporated the tonic excitatory drives to select neuron populations on both sides of the spinal network in order to simulate the brainstem input to the spinal cord activated by electrical stimulation (see Methods and our previous publications [43,44,45]). The brainstem drives in the model activated the RGs, the commissural CINe populations projecting to the contralateral V1 and V1-1 populations, and the inhibitory Ini populations (see Figure 5A). The activation of the RGs set up the locomotor oscillations in the spinal network, whereas the activation of the V1 and V1-1 populations (via commissural CINe populations) influenced the oscillation frequency.

This model was also able to generate spinal cord oscillations with alternating rhythmic activity of the F and E half-centers on each side and alternating activity of the left and right RGs (Figure 5B1). The notable differences in operation of this model (Figure 5B1) in comparison to the model of drug-induced fictive locomotion (Figure 1B1) were the absence of tonic (ipsilateral flexor) components in the activity of V1-1 population and the presence of tonic component in the activity of Ini populations. These differences made the oscillation frequency in the brainstem stimulation-evoked fictive locomotion more dependent on the activity of V1 populations than on the activity of V1-1 populations, as this was in the model of drug-induced fictive locomotion.

### 2.5. Simulating the Effects of Optogenetic Silencing and Activation of V1 Neurons on the Locomotor Rhythm and Pattern

In both versions of our model, optogenetic (light-induced) hyperpolarization (silencing) and depolarization (activation) of V1 neurons were simulated by increasing the conductance of archaerhodopsin ($g_{\mathrm{Ar}}$) or channelrhodopsin ($g_{\mathrm{Ch}}$) channels, respectively, which in control conditions were set to 0 (see Materials and Methods).

#### 2.5.1. Silencing and Activation of V1 Neurons during Drug-Induced Fictive Locomotion in the Intact Cord

**Figure 6:**
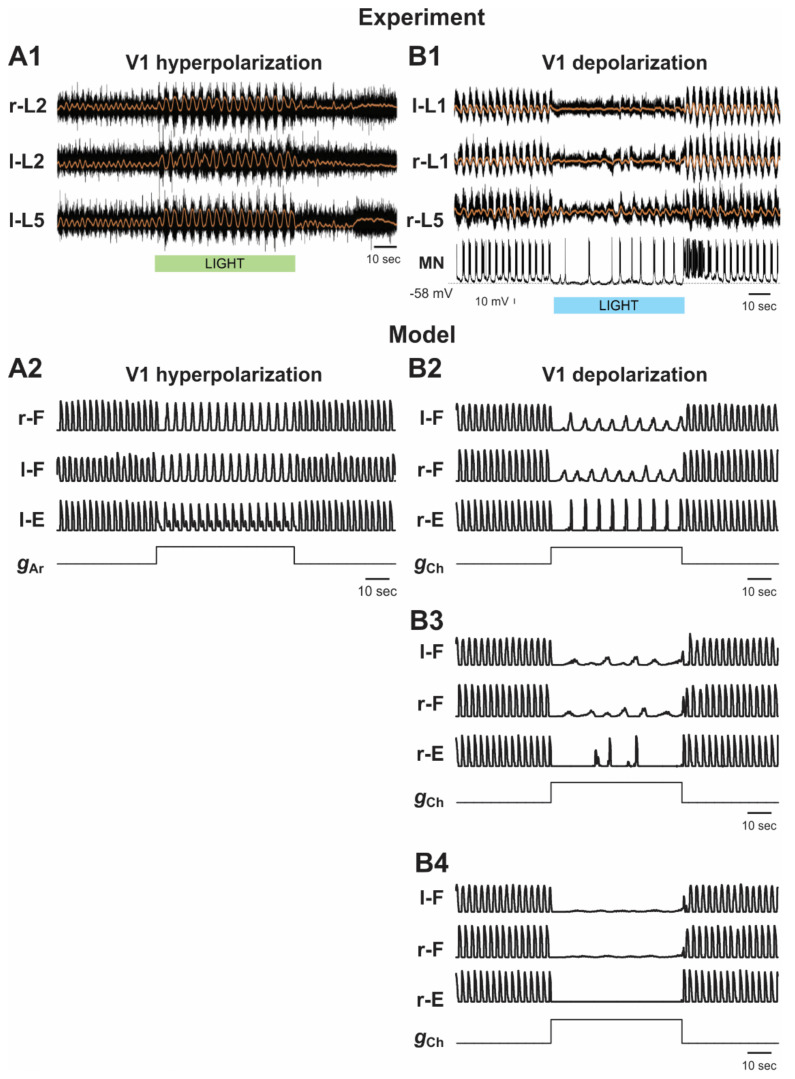
Effects of the selective hyperpolarization and depolarization of V1 neurons on drug-induced fictive locomotion. (**A1**,**B1**) Locomotor-like activity recorded from left and right lumbar roots (black traces) during drug-induced fictive locomotion are shown overlapped with the slow potentials obtained by low pass filtration of raw traces (orange lines) before, during, and after application of hyperpolarizing (**A1**) and depolarizing (**B1**) light stimuli. In (**B1**), the activity of a flexor motoneuron (MN) is also shown. The corresponding light stimulations (duration 60 s) are shown by green (**A1**) and blue (**B1**) bars. Locomotion was induced by application of 5 µM NMDA, 10 µM 5-HT, and 50 µM DA. (**A1)** reproduced from Falgairolle and O’Donovan [35], their Figure 5A, under CC-BY license. (**B1**) reproduced from Falgairolle and O’Donovan [36], their Figure 2A, under CC-BY license. (**A2**,**B2**–**B4**) The results of our simulation of hyperpolarization (**A2**) and depolarization (**B2**–**B4**) of all V1 neurons during drug-induced fictive locomotion in the intact cord. All traces show integrated activity profiles of the F and E half-centers before, during, and after increasing $g_{\mathrm{Ar}}$ or $g_{\mathrm{Ch}}$ (lower stepwise trace) to simulate V1 hyperpolarization and depolarization, respectively. In (**A2**), $g_{\mathrm{Ar}}$ was increased to 7.0 mS/cm^2^. In (**B2**–**B4**), $g_{\mathrm{Ch}}$ was increased to 0.7, 0.95, and 1.0 mS/cm^2^, respectively. *α* = 0.16 in (**A2**) and 0.15 in (**B2**–**B4)**. l—left; r—right.

Hyperpolarization/silencing of V1 neurons. Optogenetic silencing or hyperpolarization of V1 neurons applied during drug-induced fictive locomotion in the intact cord of neonatal mouse was shown to significantly reduce the frequency of generated oscillations [32,35] (see an example in Figure 6A1).

In our model, the selective silencing of V1 and V1-1 populations independently had different effects on model performance and oscillation frequency. Hyperpolarization (silencing) of neurons in the V1-1 populations eliminated inhibition of each F half-center by the corresponding E half-center (Figure 1A and Figure 2), leading to shortening of the flexor interburst interval (the duration of extension) in each RG and hence to acceleration of the rhythm. In contrast, hyperpolarization or silencing of neurons in the V1 populations led to disinhibition of the ipsilateral Ini populations (Figure 1A and Figure 2), enhancing their activity and providing tonic inhibition of the F half-centers (Figure 1B2), leading to the deceleration (slowing down) of the generated rhythmic activity. However, the effect of silencing the V1 populations in the model was much stronger than the accelerating effect of silencing the V1-1 populations. Therefore, the resultant effect of silencing of both V1 and V1-1 populations together was a slowing down of the rhythmic activity (Figure 1B2 and Figure 6A2), which was consistent with the corresponding experimental data (Figure 6A1).

Depolarization/activation of V1 neurons. The results of optogenetic depolarization of V1 neurons applied during drug-induced fictive locomotion in the intact cord were not that clear and rather were surprising [36]. Seemingly, the effect of V1 neuron depolarization would be expected to be opposite to the effect of their hyperpolarization, as described above, i.e., the expected effect would be acceleration of drug-induced oscillation. However, a previous study [33] reported that optogenetic activation of V1 neurons silenced the ventral root activity, which could be either at the level of the RG or at the motoneuron level [36,42]. In the experiments of Falgairolle and O’Donovan [36], the rhythmic activity was suppressed at the beginning of light stimulation followed by a recovery of low-frequency, irregular bursting (Figure 6B1). The general conclusion of the authors was that V1 depolarization in this case most often led to the reduction of oscillation frequency.

The results of our simulation of the effects of V1 neuron depolarization during drug-induced fictive locomotion were qualitatively consistent with the above experimental studies and were dependent on the value of depolarization defined by $g_{\mathrm{Ch}\;}$(Figure 6B2–B4). At a relatively weak depolarization of V1 neurons, the model demonstrated a slowing of the generated oscillations (Figure 1B3 and Figure 6B2). In all neurons of V1-1 populations, this depolarization summated with the depolarization produced by drugs and by the contralateral excitatory input from the CINe population and the drive, which together strongly activated the V1-1 populations, inhibiting the F half-centers of each RG, hence reducing the amplitude and frequency of flexor bursts and slowing rhythmic activity in the entire network.

Further increases of simulated optogenetic depolarization of V1 neurons led to irregular bursting (Figure 6B3) and, finally, to a full suppression of the activity in each RG, due to overexcitation of ipsilateral V1-1 and V1 populations (Figure 6B4), which was previously shown in our model [42] and corresponds to the experimental data of Britz et al. [33].

#### 2.5.2. Silencing and Activation of V1 Neurons during Drug-Induced Fictive Locomotion in the Isolated Hemicord

**Figure 7:**
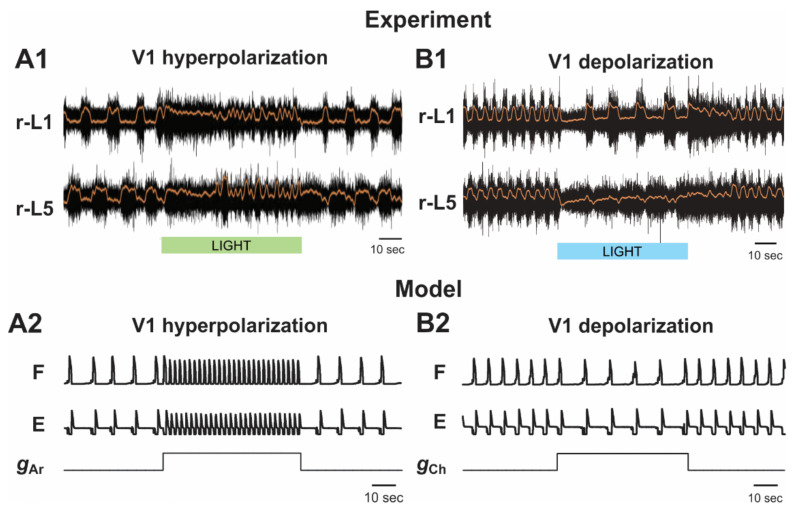
Effects of the selective hyperpolarization and depolarization of V1 neurons on drug-induced fictive locomotion in the isolated hemicord. (**A1**,**B1**) Fictive locomotor activity recorded from lumbar roots (black traces) of isolated hemicord overlapped with the slow potentials obtained by low pass filtration of raw traces (orange lines). The V1 hyperpolarizing and V1 depolarizing light stimulations (duration 60 s) are shown by green (in **A1**) and blue (in **B1**) bars, respectively. (**A1**) reproduced from Falgairolle and O’Donovan [35], their Figure 9A, under CC-BY license. (**B1**) reproduced from Falgairolle and O’Donovan [36], their Figure 3A, under CC-BY license. (**A2**) and (**B2**) The results of our simulation of hyperpolarization (**A2**) and depolarization (**B2**) of all V1 neurons during drug-induced fictive locomotion in the hemicord. All traces show integrated activity profiles of the F and E populations before, during, and after increasing $g_{\mathrm{Ar}}$ or $g_{\mathrm{Ch}}$ (lower stepwise trace) to simulate V1 hyperpolarization and depolarization, respectively. In (**A2**), $g_{\mathrm{Ar}}$ was increased to 7.0 mS/cm^2^. In (**B2**), $g_{\mathrm{Ch}}$ was increased to 0.7 mS/cm^2^. α = 0.18 in (**A2**) and 0.2 in (**B2**).

Falgairolle and O’Donovan have previously demonstrated that optogenetic hyperpolarization of V1 neurons in the isolated mouse hemicord produces an increase in the frequency of drug-induced fictive locomotion [35] (Figure 7A1), whereas their optogenetic activation slows down these oscillations [36] (Figure 7B1).

As described above, the V1 population in our hemicord model was always silent (Figure 3). Hyperpolarization of neurons in the V1-1 population silenced them as well (Figure 3B2). Silencing the V1-1 population eliminated inhibition of the F half-center by the E half-center, leading to the significant increase in the frequency of the oscillations in the network that were generated intrinsically by the F half-center (Figure 3B2 and Figure 7A2), which was consistent with experimental studies [35] (Figure 7A1).

Depolarization of V1 neurons in the hemicord model produced the opposite effect. It increased the activity of the V1-1 population, hence increasing inhibition of the F half-center by this population, leading to a slowing down of the generated oscillations (Figure 3B3 and Figure 7B2), which was also consistent with the corresponding experimental data [36] (Figure 7B1).

#### 2.5.3. Activation and Silencing of V1 Neurons during Brainstem Stimulation-Evoked Fictive Locomotion

**Figure 8:**
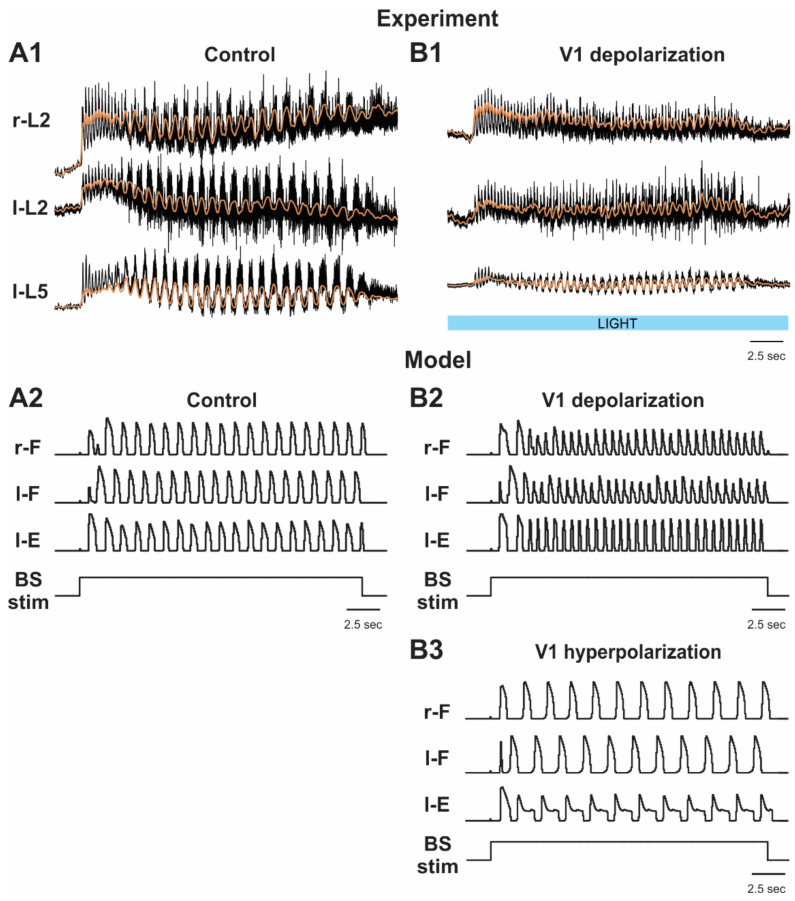
Effects of selective depolarization and hyperpolarization of V1 neurons on fictive locomotion evoked by brainstem stimulation. (**A1**,**B1**) Fictive locomotor activity recorded from left and right lumbar roots (black traces) overlapped with the slow potentials obtained by low pass filtration of the raw traces (orange lines) are shown before (**A1**; control) and during (**A2**) application of depolarizing light stimuli. The light duration is shown by the blue bar. Reproduced from Falgairolle and O’Donovan [36], their Figure 6A1,A2, under CC-BY license. (**A2**,**B2**,**B3**) The results of our simulations of locomotor oscillations evoked by brainstem stimulation (BS stim; bottom traces) in control conditions (**A2**) and following simulated light-induced depolarization (**B2**) and hyperpolarization (**B3**) of all V1 neurons. In these simulations, $w_{dF\;}$ = 0.4, $w_{dE\;}$ = 0.48, $w_{d\mathrm{CINe}\;}$ = 0.75, and $w_{d\mathrm{Ini}\;}$ = 2. All traces show the integrated activity profiles of the F and E half-centers. V1 neuron depolarization (**B2**) and hyperpolarization (**B3**) was simulated by increasing $g_{\mathrm{Ch}}$ or $g_{\mathrm{Ar}}$, respectively. In (**B2**), $g_{\mathrm{Ch}}$ was increased to 0.7 mS/cm^2^. In (**B3**), $g_{\mathrm{Ar}}$ was increased to 7 mS/cm^2^. l—left; r—right.

Figure 8B1 shows examples of experimental recordings of activity recorded from lumbar roots during fictive locomotion evoked by electrical stimulation of the brainstem before and during optogenetic activation (depolarization) of V1 neurons [36]. In these experiments, the light-induced depolarization of V1 neurons caused a significant acceleration of the rhythm. This effect was surprisingly opposite to the effect of V1 neuron activation in the case of drug-induced fictive locomotion, in which activation of the V1 neurons affected the regularity of the rhythm and slowed it down (see Figure 6B1).

This difference was reproduced in our simulation of brainstem stimulation-evoked fictive locomotion. Simulation of depolarization of all V1 neurons in this model resulted in an increase in oscillation frequency (Figure 5B3 and Figure 8B2), which was opposite to what was observed following activation of all V1 neurons in the model of drug-induced locomotion, where this activation slowed the rhythm or suppressed oscillations (Figure 1B3 and Figure 6B2–B4).

The model was also used to simulate and hence to predict the effects of V1 hyperpolarization on the frequency of fictive locomotor oscillations evoked by brainstem stimulation. As shown in Figure 5B2 and Figure 8B3, the model predicted that hyperpolarization or silencing of V1 neurons in this preparation should lead to a slowing of oscillations evoked by brainstem stimulation, which in the model resulted from an increasing inhibition of F half-centers of the RGs by the Ini populations (Figure 5B2).

## 3. Discussion

### 3.1. Modeling the Locomotor Circuits in the Mammalian Spinal Cord

Although the present study was mainly focused on V1 neurons, the importance of the proposed model would be low if the model could only reproduce experimental data from V1 neuronal manipulations. Our ultimate goal has always been to develop a generalized model that would be consistent with all available data sets obtained in different laboratories under different experimental conditions. The network architecture of the present model was based on our previous models [31,40,41,42] that could reproduce experimental data from multiple studies using selective removal, silencing, inhibition, or activation of different types of genetically identified spinal interneurons. Using these previous models as a basis, we were very careful and tried to make sure that the novel, updated model would be still able to reproduce prior experimental data. Therefore, the present model can also reproduce the previously described effects of genetic ablation of V0_V_ or/and V0_D_ commissural interneurons [27], silencing of ipsilaterally projecting V1 or/and V2b neurons in the intact cord and hemicord [32], and both unilateral and bilateral optogenetic stimulation of V3 neurons [31]. In addition, the present model can reproduce and provide explanations for the results of recent studies concerning the effects of selective inhibition [35] and activation [36] of V1 neurons during fictive locomotion evoked under different conditions. Therefore, despite the relative complexity of the model and many tunable parameters, the number of different experimental phenomena that the final model can reproduce provides certain validation of the proposed model at this stage.

### 3.2. Two Distinct Types of Populations of V1 Neurons within the Locomotor Spinal Circuitry and Their Involvement in Flexor–Extensor Alternation and Control of Oscillation Frequency

The V1 neurons belong to a large heterogenous class of ipsilaterally-projecting inhibitory neurons that comprise multiple functionally different cell types, including Ia inhibitory interneurons and Renshaw cells mediating different forms of inhibition at the level of motoneurons [25,48,49]. In the model presented here, we consider and use the term “V1 neurons” for only the populations of V1 neurons that presumably operate within the rhythm-generating and premotor spinal circuits and are involved in flexor–extensor and left–right interactions in these circuits. In other words, the V1 populations that operate within reflex circuits, such as Ia interneurons or Renshaw cells, have not been considered.

The experimental study of Zhang et al. [32] has shown that V1 neurons (together with V2b neurons) contribute to flexor–extensor alternation in the intact cord but not in the isolated hemicord, where the selective removal of only V2b neurons led to flexor–extensor synchronization (see also our previous publications [40,42]. At the same time, the recent results of Falgairolle and O’Donnovan [35,36] suggest that V1 neurons contribute to frequency control in both the intact cord and hemicord. To make the model consistent with both sets of data, we included in the model and hence suggested that the spinal locomotor network contains two functionally distinct populations of V1 neurons.

One type of V1 neuron, designated in our model as the V1 population, is involved in the contralateral (commissural) inhibitory pathway. This pathway originates at the contralateral descending CIN population, which excites the ipsilateral V1 population that is also rhythmically inhibited by the contralateral inhibitory V0_D_ CINs. This V1 population in turn inhibits the ipsilateral E half-center. These bilaterally located V1 populations critically contribute to flexor–extensor alternation in the intact cord; they support and secure this alternation even when V2b neurons are ablated or silenced [32] (see additional explanations in [42]). In addition, these V1 populations are critically involved in the control of the frequency of fictive locomotion in the intact cord, providing disinhibition of each ipsilateral F half-center, so that activation of the V1 populations leads to an increase in the locomotor frequency.

The other type of V1 neurons, designated in the model as the V1-1 population, contributes to flexor–extensor alternation by mediating inhibition of the F half-center by the corresponding E half-center on each side of the cord. Activation of the V1-1 populations reduces the locomotor frequency, which becomes significant after hemisection, when the V1 populations become silent.

The existence of the two functionally distinct populations of V1 neurons (V1 and V1-1 types) predicted by our model now awaits experimental testing, which, considering the many identified subtypes of V1 neurons [48,49], may require substantial effort and time.

### 3.3. Different Effects of V1 Activations during Drug-Induced and Brainstem Stimulation-Evoked Fictive Locomotion

In their recent study, Falgairolle and O’Donnovan [36] reported that optogenetic depolarization of V1 neurons had surprisingly different effects on fictive locomotion induced by drugs versus fictive locomotion evoked by brainstem stimulation. In the former case, the depolarization of V1 neurons slowed down the generated rhythmic activity, whereas in the latter case, their depolarization resulted in the acceleration of this activity. Our model reproduces and proposes an explanation for this difference based on the distinct roles of V1 and V1-1 populations in the control of fictive locomotion frequency as described in Section 2.4 and above. The oscillation frequency in the brainstem stimulation-evoked fictive locomotion is more dependent on the activity of V1 populations that inhibit ipsilateral Ini populations and hence disinhibit the F half-centers of both RGs, producing an increase of oscillation frequency. In the case of drug-induced fictive locomotion, the frequency is mainly defined by the activity of V1-1 populations that inhibit the F half-centers of both RGs and either slow or fully suppress the generated oscillations. Therefore, the observed opposite effects of V1 depolarization in the two preparations can be reproduced in the same network and do not require different network organization as was suggested by Falgairolle and O’Donnovan [36].

This conclusion is confirmed by our simulations of the effect of V1 neuron hyperpolarization. In both above cases (i.e., independent of the way the fictive locomotion was produced in the model), the hyperpolarization of V1 resulted in the reduction in the frequency of locomotor oscillations. This conclusion is already consistent with the experimental studies of drug-induced locomotion [35] (see Figure 7A). The frequency reduction by the optogenetic hyperpolarization or silencing of V1 neurons in the case of brainstem stimulation-evoked fictive locomotion predicted by our simulation awaits experimental testing in the future.

### 3.4. Reduction of Frequency of Drug-Induced Fictive Locomotion following Midsagittal Hemisection

Studies utilizing isolated cords from neonatal rats showed that complete sagittal transections made parallel to the midline allowed for the generation of drug-evoked locomotor oscillations in a remaining larger part of the cord [50,51]. The frequency of these oscillations was reduced, and the reduction in frequency was related to how close/far the section was from the midline. Many previous studies have demonstrated that the drug-induced fictive locomotor activity can be evoked in an isolated hemicord of neonatal mouse after the complete midsagittal transection [32,35,36,46,47]. The common observation from these studies is that midsagittal hemisection results in the significant reduction of the frequency of drug-evoked oscillations.

Understanding the nature of the frequency reduction following midsagittal cord transection can shed light on the mechanisms, pathways, and neurons involved in the left–right interactions in the spinal cord. However, this phenomenon has not been explicitly investigated so far. Logically, this phenomenon should be connected with some commissural pathways from the contralateral side that affect the operation of each rhythm generating circuit in the intact cord but are eliminated by midsagittal transection. One could suggest that such commissural pathways can be mediated by the well-known V0 (V0_V_ and V0_D_) CINs. The genetic ablation of V0_V_ CINs disturbed left–right alternation (promoting a switch to left-right synchronization) at moderate/high frequencies, and the ablation of both V0 CIN subtypes led to left–right synchronization and hopping-like activity at all locomotor frequencies [10,27]. However, the ablation of V0 CINs mediating left–right inhibition and the resultant switching from left–right alternation to left–right synchronization in isolated cords did not show visible changes in the range of frequencies observed during drug-evoked fictive locomotion [27]. Therefore, despite the attractiveness of this idea, it is very unlikely that the reduction in the frequency of locomotor activity following midsagittal hemisection results from the removal of V0 CINs.

Our model proposed an explanation for frequency reduction following hemisection based on the critical role of V1 neurons. In our model, RGs on each side (particularly the F half-centers) are suppressed by local inhibitory neurons (Ini populations in the model), which in turn are inhibited by the V1 populations that receive tonic excitation from the contralateral side of the cord. Such contralateral excitation can be provided by populations of descending CINs located in the contralateral hemicord that receive brainstem drive and/or are activated by drugs (in the case of drug-induced locomotion), such as the CINe populations in our model. Such CIN populations have been previously described in rats and cats [52,53,54,55,56,57,58]. As a result, the V1 populations, while receiving the contralateral excitation, inhibit the Ini populations and hence disinhibit both RGs, allowing them to generate high frequency oscillations. In turn, the midsagittal hemisection eliminates the contralateral excitation to the V1 populations, making them silent, and hence the RG in the isolated cord operates under constant inhibition of the Ini population that significantly reduces the frequency of generated oscillations in the hemicord.

We have also used our models to simulate the changes in the frequency of locomotor oscillations in the intact cord and hemicord with an increase in neuronal excitability of all modelled neurons, which presumably imitates the increase of the neuroactive drug concentration, such as NMDA. Our simulations predicted the monotonic increase of oscillation frequencies in both preparations with a steeper frequency increase in case of the intact cord. Our experimental testing provided qualitative support of our modeling predictions.

### 3.5. Model Limitations

Our ultimate goal was to develop a united model that would be consistent with a large body of data obtained in multiple laboratories under different experimental conditions. Major parts of the network architecture used in the present model were based on our previous models [31,40,41], which are able to reproduce experimental data from multiple studies. At the same time, considering the complexity of the proposed model and current insufficiency of experimental data, the proposed model has obvious limitations. Particularly, the model was mostly based on data from experimental studies performed *in vitro* using isolated neonatal mouse spinal cords, with the motor outputs recorded from ventral roots that characterized the integrated flexor and extensor activities on each side but did not allow for the analysis of the activity of individual motoneuron types. These experiments did not consider the potential effects of sensory afferent stimulation. Therefore, the present model focused exclusively on central interactions within the spinal cord without considering spinal circuits operating below the rhythm-generating and left–right coordinating circuits. The model does not include motoneurons, and we assume that the motor output (activity recorded from the lumbar roots) simply reproduces the output activity in rhythm generating circuits. Therefore, different pattern formation circuits, circuits involved in the processing of sensory feedback, and reflex circuits, including those mediating by Ia and Ib interneurons, Renshaw cells, and motoneurons [47,59,60,61,62,63,64], were not included in the model. These circuits play an important role in the operation of the spinal locomotor network. In future modeling studies, we will focus on sequential reduction of the above limitations.

Nevertheless, here we took advantage of computational modeling to propose and analyze the role of distinct types of V1 neuron populations, their involvement in different ipsilateral and contralateral interactions in the spinal cord, and their contribution to the control of the frequency of locomotor oscillations. Several predictions concerning the organization of spinal V1 neurons and their involvement in different ipsilateral and contralateral interactions within the spinal cord were made that await experimental testing.

## 4. Materials and Methods

### 4.1. Modeling Methods

#### 4.1.1. Modeling Single Neurons and Neuronal Populations

All neurons were simulated in the Hodgkin–Huxley style as single-compartment models. The F half-centers in the model have 200 neurons, while all other populations have 50 or 100 neurons (see Table 1). The neurons in the F and E half-centers incorporate a slowly inactivating persistent sodium current (*I*_NaP_) and are connected by excitatory synaptic connections that allow them to generate synchronized populational bursting activity in a certain range of an external brainstem drive. The membrane potential, *V*, in neurons of the left and right F and E populations is described by the following differential equation

(1) $$C\times \frac{dV}{dt}=-I_{\mathrm{Na}}-I_{\mathrm{NaP}}-I_{K}-I_{L}-I_{\mathrm{SynE}}-I_{\mathrm{SynI}},$$

where *C* is the membrane capacitance, and *t* is time.

The neurons in all other populations represent simple spiking neurons, and their neuron membrane potential is described as follows:

(2) $$C\times \frac{dV}{dt}=-I_{\mathrm{Na}}-I_{\mathrm{NaP}}-I_{K}-I_{L}-I_{\mathrm{SynE}}-I_{\mathrm{SynI}},$$

The ionic currents in Equations (1) and (2) are described as follows:

(3) $$I_{\mathrm{Na}}={\bar{g}}_{\mathrm{Na}}\times m_{\mathrm{Na}}^{3}\times h_{\mathrm{Na}}\times \left(V-E_{\mathrm{Na}}\right);\;I_{\mathrm{NaP}}={\bar{g}}_{\mathrm{NaP}}\times m_{\mathrm{NaP}}\times h_{\mathrm{NaP}}\times \left(V-E_{\mathrm{Na}}\right);\;I_{K}={\bar{g}}_{K}\times m_{K}^{4}\times \left(V-E_{K}\right);\;I_{L}=g_{L}\times \left(V-E_{L}\right);\;I_{\mathrm{Ar}}=g_{\mathrm{Ar}}\times \left(V-E_{\mathrm{Ar}}\right);\;I_{\mathrm{Ch}}=g_{\mathrm{Ch}}\times \left(V-E_{\mathrm{Ch}}\right),$$

where $\;I_{\mathrm{Na}}$ is the fast sodium current with maximal conductance ${\bar{g}}_{\mathrm{Na}}$; $I_{\mathrm{NaP}}$ is the persistent (slowly inactivating) sodium current with maximal conductance ${\bar{g}}_{\mathrm{NaP}}$ (included only in RG neurons); $I_{K}$ is the delayed-rectifier potassium current with maximal conductance ${\bar{g}}_{K}$; $I_{L}$ is the leakage current with constant conductance $g_{L}$; $I_{\mathrm{Ar}}$ and $I_{\mathrm{Ch}}$ are the archaerhodopsin and channelrhodopsin currents with the conductances $g_{\mathrm{Ar}}$ and $g_{\mathrm{Ch}}$, respectively, that were included in V1 neurons only to simulate light application. $E_{\mathrm{Na}}$, $E_{K}$, $E_{L}$, $E_{\mathrm{Ar}}$, and $E_{\mathrm{Ch}}$ are the reversal potentials for the sodium, potassium, leakage, archaerhodopsin, and channelrhodopsin currents, respectively. Variables *m* and *h* with indexes indicating ionic currents are the activation and inactivation variables, respectively, of the corresponding ionic channels. The values of maximal conductances for ionic currents and initial average values for the leakage reversal potential in each neuron population are specified in Table 1. The type and values for $g_{\mathrm{Ar}}$ and $g_{\mathrm{Ch}}$ in specific simulations are indicated in the corresponding figure legends.

**Table 1:** Number of neurons and neuron parameters in different populations.

Neuron Type	N, Number of Neurons	${\bar{g}}_{Na}$, mS/cm^2^	${\bar{g}}_{NaP}$, mS/cm^2^	${\bar{g}}_{K}$, mS/cm^2^	$g_{L}$, mS/cm^2^	$E_{L}$, mV
F	200	25	0.75 (±0.00375)	2	0.07	−76.8 (±0.77)
E	100	25	0.75 (±0.00375)	2	0.07	−72 (±0.72)
V2b	100	10		5	0.1	−80.4 (±1.68)
V1-1	100	10		5	0.1	−90 (±1.8)
V1	100	10		5	0.1	−90 (±1.8)
Ini	50	10		5	0.1	−60 (±1.2)
Ini1	50	10		5	0.1	−76.8 (±1.57)
V2a	50	40		5	0.8	−72.6 (±1.45)
V_0V_	50	10		5	0.1	−74.4 (±1.5)
V_0D_	50	10		5	0.1	−81.6 (±2.45)
V3	100	10		5	0.1	−81.6 (±2.45)
CINe	100	10		5	0.1	−60 (±2.04)

Activation *m* and inactivation *h* of voltage-dependent ionic channels (e.g., Na, NaP, and K) in Equation (3) are described by the following differential equations:

(4) $$\tau_{mi}\left(V\right)\times \frac{d}{dt}m_{i}=m_{\infty i}\left(V\right)-m_{i};\;\tau_{hi}\left(V\right)\times \frac{d}{dt}h_{i}=h_{\infty i}\left(V\right)-h_{i},$$

where $m_{\infty i}\left(V\right)$ and $h_{\infty i}\left(V\right)$ define the voltage-dependent steady-state activation and inactivation of the channel *i*, respectively, and $\tau_{mi}\left(V\right)$ and $\tau_{hi}\left(V\right)$ define the corresponding time constants. Activation of the sodium channels are considered instantaneous ($\tau_{m\mathrm{Na}\;}$ =$\;\tau {\;}_{m\mathrm{NaP}}\;$= 0). The expressions for channel kinetics are given in Table 2.

The synaptic excitatory ($I_{\mathrm{SynE}}$ with conductance $g_{\mathrm{SynE}}$ and reversal potential $E_{\mathrm{SynE}}$) and inhibitory ($I_{\mathrm{SynI}}$ with conductance $g_{\mathrm{SynI}}$ and reversal potential $E_{\mathrm{SynI}})$ currents are described as follows:

(5) $$I_{\mathrm{SynE}}=g_{\mathrm{SynE}}\times \left(V-E_{\mathrm{SynE}}\right);\;I_{\mathrm{SynI}}=g_{\mathrm{SynI}}\times \left(V-E_{\mathrm{SynI}}\right),$$

where $g_{\mathrm{SynE}}$ and $g_{\mathrm{SynI}}$ are equal to zero at rest and are activated by the excitatory or inhibitory inputs, respectively, to neuron *i*:

(6) $$g_{S\mathrm{ynE}i}\left(t\right)={\bar{g}}_{E}\times \underset{j}{\sum }S\left\{w_{ji}\right\}\times \underset{t_{kj}<t}{\sum }exp(-\left(t-t_{kj}\right)/\tau_{\mathrm{SynE}});$$

(7) $$g_{\mathrm{SynI}i}\left(t\right)={\bar{g}}_{I}\times \underset{j}{\sum }S\left\{-w_{ji}\right\}\times \underset{t_{kj}<t}{\sum }exp(-\left(t-t_{kj}\right)/\tau_{\mathrm{SynI}}),$$

where *S*{*x*} = *x*, if *x* ≥ 0, and 0 if *x* < 0. Each spike arriving to neuron *i* in a target population from neuron *j* in a source population at time *t_kj_* increases the excitatory synaptic conductance by ${\bar{g}}_{E}\times w_{ij}$ if the synaptic weight $w_{ij}$> 0, or increases the inhibitory synaptic conductance by $-{\bar{g}}_{I}\times w_{ij}$ if the synaptic weight $w_{ij}$ < 0. ${\bar{g}}_{E}$ and ${\bar{g}}_{I}$ define an increase in the excitatory or inhibitory synaptic conductance, respectively, produced by one arriving spike at |$w_{ij}$| = 1. $\tau_{\mathrm{SynE}}$ and $\tau_{\mathrm{SynI}}$ are the decay time constants for $g_{\mathrm{SynE}}$ and $g_{\mathrm{SynI}}$, respectively.

The following general neuronal parameters were assigned: C = 1*μ*F·cm^−2^; $E_{\mathrm{Na}}$ = 55 mV; $E_{\mathrm{Ka}}$ = − 80 mV; $E_{\mathrm{Ar}}$= −80 mV; $E_{\mathrm{Ch}}$ = −10 mV; $E_{\mathrm{SynE}}$ = −10 mV; $E_{\mathrm{SynI}}$ = −70 mV; ${\bar{g}}_{E}\;$=${\bar{\;g}}_{I}\;$=0.05 mS/cm^2^; $\tau_{\mathrm{SynE}}\;$= $\tau_{\mathrm{SynI}}$ = 5 ms.

Heterogeneity of neurons within each population was provided by random distributions of the leakage reversal potentials $E_{L}$ (see mean values ± SD for each population in Table 1) and initial conditions for the values of membrane potential and channel kinetics variables. The values of $E_{L}$ and all initial conditions were assigned prior to simulations from the defined average values and variances using a random number generator, and a settling period of 10–200 s was allowed in each simulation.

Random synaptic connections between the neurons of interacting populations were assigned prior to each simulation based on an assigned probability of connection, *p*, so that if a population *A* is assigned to receive an excitatory (or inhibitory) input from a population *B*, then each neuron in population *A* receives the corresponding synaptic input from each neuron in population *B* with the probability *p*{*A*, *B*}. If *p*{*A*, *B*} < 1, a random number generator is used to define the existence of each synaptic connection; otherwise, if *p*{*A*, *B*} = 1, each neuron in population *A* receives synaptic input from each neuron of population *B.* Values of synaptic weights ($w_{ij}$) are also set using a random number generator based on the average values of these weights, w, and the variances, which are defined as 5% of w for excitatory connections (w > 0) and 10% of w for inhibitory connections (w < 0). The average weights and probabilities of connections are specified in Table 3.

#### 4.1.2. Generation of Rhythmic Activity in the Model and Application of Photostimulation

To simulate the experimental methods of inducing locomotor activity in the isolated spinal cord, we considered two methods of evoking of the rhythmic activity in the model. In the first version of the model (Figure 1A and Figure 3A), to simulate activation of rhythmic oscillations by application of neuroactive drugs, we introduced a parameter, α, that defined the average neuron excitation in each population *i*: ${\bar{E}}_{\mathrm{Li}}={\bar{E}}_{\mathrm{LiO}}\cdot \left(1-\alpha \right)$, where ${\bar{E}}_{\mathrm{LiO}}$ represented the baseline value of the leakage reversal potential in the population at α = 0. The enhancement of neuronal excitation in the cord in response to application of neuroactive drugs was simulated by increasing α.

In the second version of the model (Figure 5A), to simulate the evoking of locomotor oscillations by stimulation of the brainstem, we incorporated in the model a tonic excitatory drive to specific neuron populations, such as F, E, CINe, and Ini populations in each side. For these populations, Equation (6) was modified as follows:

(8) $$g_{S\mathrm{ynE}i}\left(t\right)={\bar{g}}_{E}\times \underset{j}{\sum }S\left\{w_{ji}\right\}\times \underset{t_{kj}<t}{\sum }exp(-\left(t-t_{kj}\right)/\tau_{\mathrm{SynE}})+{\bar{g}}_{Ed}\cdot S\left\{w_{di}\right\}\cdot d.$$

In Equation (8), the excitatory synaptic conductance has two terms: one describing the effects of excitatory inputs from other neurons in the network, as in Equation (6), and the other describing effects of inputs from the external (brainstem) excitatory drive (see also [60,61]. In the second terms of Equation (8), ${\bar{g}}_{Ed}$ is the parameter defining the increase in the excitatory synaptic conductance, produced by external input drive *d* = 1 with a synaptic weight of |$w_{d}$| = 1; ${\bar{g}}_{Ed}\;$= 0.05 mS/cm^2^. The values of $w_{di}$ are indicated in the corresponding figure legends.

To simulate the effect of photostimulation, we selectively depolarized or hyperpolarized all V1 neurons by increasing the corresponding rhodopsin current conductance ($g_{\mathrm{Ar}}$ or $g_{\mathrm{Ch}}$), which was set to 0 in control conditions. The values of $g_{\mathrm{Ar}}$ or $g_{\mathrm{Ch}}$ in these simulations were increased to $g_{\mathrm{Ar}}$ = 7 mS/cm^2^ and $g_{\mathrm{Ch}}$ = 0.7 mS/cm^2^, respectively, if not indicated otherwise.

#### 4.1.3. Computer Simulations

All simulations were performed using the custom neural simulation package NSM 2.5.6. The simulation package and model configuration files to create the simulations presented in the paper are available at https://github.com/RybakLab/nsm (accessed on 20 March 2022). The simulation package was previously used for the development of several spinal cord models [31,37,38,39,44,57,58,59,61]. Differential equations were solved using the exponential Euler integration method with a step size of 0.1 ms.

### 4.2. Experimental Materials and Methods

Procedures for experiments performed related to Figure 4 were approved by the Institutional Animal Care and Use Committee at Drexel University and followed the guidelines of the National Institutes of Health for laboratory animal welfare.

Spinal cords were isolated from 5 mice at postnatal day 1–3. Briefly, mice were decapitated and eviscerated. Spinal cords were then removed in a cold dissecting solution containing in mM: 111 NaCl, 3 KCl, 11 glucose, 25 NaHCO_3_, 3.7 MgSO_4_, 1.1 KH_2_PO_4_, and 0.25 CaCl_2_, and aerated with 95%O_2_/5%CO_2_. Cords were then transferred into room temperature artificial cerebrospinal fluid solution containing in mM: 111 NaCl, 3 KCl, 11 glucose, 25 NaHCO_3_, 1.3 MgSO_4_, 1.1 KH_2_PO_4_, and 2.5 CaCl_2_, aerated with 95%O_2_/5%CO_2_. Glass suction electrodes were used to record from lumbar (L) ventral roots, typically L1, L2, or L3 and L4 or L5 on left and right sides of the cord. Signals were amplified 1000× and bandpass filtered (10–1000 Hz) on a MA 102 amplifier (Zoological Institute, University of Cologne, Germany). Signals were digitized at 100 kHz with an Axon Digidata 1550A and acquired using pClamp software.

Locomotor-like activity was initially induced by application of 7 μM N-methyl-D-aspartic acid (NMDA, Sigma) and 8 μM serotonin creatinine sulfate monohydrate (5-HT, Sigma) in all cases. Following the observation of stable locomotor-like activity, drugs were washed off and then reapplied beginning at high concentrations of NMDA (9–12 μM) and decreased in intervals of 2–3 μM of NMDA, all in the presence of 8 μM 5-HT. Each concentration was applied for a minimum of 10 min. The spinal cord was then hemisected in ACSF, and NMDA and 5-HT were reapplied following the same procedure. In most cases (4/5), roots from both left and right sides were recorded. Recording of a continuous 5 min time span at each concentration was used for analysis. Frequency was determined by dividing the number of bursts in a single root in 5 min by 300 s. Since 9 μM NMDA was a concentration applied in all intact cords, data were normalized to the frequency of intact locomotor-like activity at 9 μM NMDA. Normalized data from 3, 5, 7, and 9 μM NMDA are displayed, but not all concentrations were tested in each of the 5 cords.

## Figures and Tables

**Table 2:** Steady state activation and inactivation variables and time constants for voltage-dependent ionic channels.

Ionic Channels	*m_∞_*(*V*) and *h_∞_*(*V*), *V* in mV	*τ_m_*(*V*) and *τ_h_*(*V*), ms
Fast sodium, Na	$m_{\infty \mathrm{Na}}=\left(1+exp(-\left(V+34\right)/7.8\right){)}^{-1}$	$\tau_{m\mathrm{Na}}=0$
	$h_{\infty \mathrm{Na}}=\left(1+exp(\left(V+55\right)/7\right){)}^{-1}$	$\tau_{h\mathrm{Na}}=20/\left(exp(\left(\left(V+50\right)/15\right)+exp(\left(-\left(V+50\right)/16\right)\right)$
Persistent sodium, NaP	$m_{\infty \mathrm{NaP}}=\left(1+exp(-\left(V+47.1\right)/3.1\right){)}^{-1}$	$\tau_{m\mathrm{NaP}}=0$
	$\;h_{\infty \mathrm{NaP}}=\left(1+exp(\left(V+60\right)/6.5\right){)}^{-1}$	$\tau_{h\mathrm{NaP}}=18000/cosh(\left(V+60\right)/13)$
Potassium delayed rectifier, K	$m_{\infty K}=\left(1+exp(-\left(V+28\right)/4\right){)}^{-1}$	$\tau_{mK}=3.5/cosh\left(\left(V+40\right)/40\right)$
	*h*_K_ = 1	

**Table 3:** Average weights (w) and probabilities (*p*) of synaptic connections between populations.

Source Population	Target Populations
F	i-F * (0.0075, *p* = 0.1) i-V2b (0.5, *p* = 0.1) i-V2a (0.3, *p* = 0.1) i-V0_D_ (0.6, *p* = 0.1)
E	i-E (0.018, *p* = 0.1) i-V1-1 (0.57, *p* = 0.1) i-V3 (0.5, *p* = 0.05)
V2b	i-E (−0.3, *p* = 0.1)
V1-1	i-F (−0.0165, *p* = 0.1)
V1	i-E (−0.05, *p* = 0.1) i-Ini (−0.06, *p* = 0.1)
Ini	i-F (−0.005, *p* = 0.1)
Ini1	i-F (−0.0025, *p* = 0.1)
V2a	i-V0_V_ (0.1, *p* = 0.1)
V0_V_	c-Ini (0.1, *p* = 0.1)
V0_D_	c-F (−0.02, *p* = 0.1) c-V1 (−0.07, *p* = 0.1)
V3	c-E (0.05, *p* = 0.05) c-V1-1 (0.3, *p* = 0.05)
CINe	i-V1 (0.14, *p* = 0.1) i-V1-1 (0.03, *p* = 0.1)

* Prefixes i- and c- indicate ipsi- and contralateral populations, respectively.

## Data Availability

The simulation package and model configuration file to create the simulations presented in the paper are available at https://github.com/RybakLab/nsm (accessed on 17 November 2017) and https://github.com/RybakLab/nsm/tree/master/models/ Shevtsova-2022-V1 (accessed on 20 March 2022).
